# Genome-wide analysis of genetic diversity in a germplasm collection including wild relatives and interspecific clones of garden asparagus

**DOI:** 10.3389/fpls.2023.1187663

**Published:** 2023-07-04

**Authors:** Tea Sala, Damiano Puglisi, Luisa Ferrari, Filippo Salamone, Maria Rosaria Tassone, Giuseppe Leonardo Rotino, Agostino Fricano, Alessia Losa

**Affiliations:** ^1^ Council for Agricultural Research and Economics – Research Centre for Genomics and Bioinformatics (CREA-GB), Montanaso Lombardo, LO, Italy; ^2^ Council for Agricultural Research and Economics – Research Centre for Genomics and Bioinformatics (CREA-GB), Fiorenzuola d’Arda, PC, Italy

**Keywords:** asparagus, germplasm collection, genetic diversity, genomic prediction, GWAS, breeding

## Abstract

The *Asparagus* genus includes approximately 240 species, the most important of which is garden asparagus (*Asparagus officinalis* L.), as this is a vegetable crop cultivated worldwide for its edible spear. Along with garden asparagus, other species are also cultivated (e.g., *Asparagus maritimus* L.) or have been proposed as untapped sources of variability in breeding programs (e.g., *Asparagus acutifolius* L.). In the present work, we applied reduced-representation sequencing to examine a panel of 378 diverse asparagus genotypes, including commercial hybrids, interspecific lines, wild relatives of garden asparagus, and doubled haploids currently used in breeding programs, which enabled the identification of more than 200K single-nucleotide polymorphisms (SNPs). These SNPs were used to assess the extent of linkage disequilibrium in the diploid gene pool of asparagus and combined with preliminary phenotypic information to conduct genome-wide association studies for sex and traits tied to spear quality and production. Moreover, using the same phenotypic and genotypic information, we fitted and cross-validated genome-enabled prediction models for the same set of traits. Overall, our analyses demonstrated that, unlike the diversity detected in wild species related to garden asparagus and in interspecific crosses, cultivated and wild genotypes of *A. officinalis* L. show a narrow genetic basis, which is a contributing factor hampering the genetic improvement of this crop. Estimating the extent of linkage disequilibrium and providing the first example of genome-wide association study and genome-enabled prediction in this species, we concluded that the asparagus panel examined in the present study can lay the foundation for determination of the genetic bases of agronomically important traits and for the implementation of predictive breeding tools to sustain breeding.

## Introduction

The *Asparagus* genus includes approximately 240 perennial species, the most economically important of which is garden asparagus (*Asparagus officinalis*) (Ellison, 1986). The edible and marketed parts of asparagus are the shoots, also known as spears, which possess a special taste and texture that is unique among vegetables, in addition to their high nutritional value owing to the content of several bioactive compounds that exhibit beneficial effects in both *in vivo* and *in vitro* experiments (Guo et al., 2020). Currently, China, Peru, Mexico, Germany, Spain, Italy, the United States, Japan, and France are the top worldwide producers of asparagus (FAO Statistics and Food and Agriculture Organization of the United Nations, 2020), while Peru has become the world’s leading exporter and controls two-thirds of the market share with China (Guo et al., 2020). In 2021, Italy produced approximately 45k tonnes of asparagus and devoted almost 7,500 hectares to its cultivation (FAO Statistics and Food and Agriculture Organization of the United Nations, 2020).

Owing to the mounting interest in this vegetable crop, genetic improvement programs, mainly based on mass selection, have been carried out in asparagus since the early 1970s, leading to the selection of several open-pollinated varieties like ‘Early of Argenteuil’ and ‘Mary Washington’ (Nikoloff et al., 1986). Currently, breeding programs in garden asparagus aim to improve the main morpho-agronomic traits of the spears, the plant architecture, and yield-related traits. Typical spear traits targeted in these programs include the time of emergence, pigmentation, firmness, turgidity of the apex, the opening of bracts in the apical part of the spear, caliber, and yield. Other important target traits evaluated at the plant level are flowering time; number, diameter, and length of stems; cladophyll density; and disease resistance.

Beyond its role as an important trait for the classification of asparagus, the pigmentation of spears has been correlated with anti-hyperglycemic, anti-cancer, and anti-mutagenic health benefits (You et al., 2021). Asparagus pigmentation is largely due to anthocyanins, which are one of the largest and most important groups of water-soluble pigments in the plant kingdom. These compounds accumulate in cell vacuoles and are primarily responsible for the diverse range of pigmentations observed in asparagus. The typical pigmentation of the spear in garden asparagus is uniformly green, although there are areas of the shoot (e.g., the tip and bracts) that are rich in anthocyanins, conferring a reddish or purplish color. In contrast to the classic green color enriched with pinkish hues, plants belonging to the ‘Violetto d’Albenga’ population and the variety known as ‘Purple Passion’ show an intense and uniform purplish coloration of the spears. Spear head firmness and compactness of bracts are two other important agronomic traits targeted in asparagus breeding programs to obtain highly appreciated commercial hybrids, as these traits contribute to the attainment of good and highly appreciated spear conformation and improved shelf life (Chang et al., 1987; Zhang et al., 2008). Moreover, spear head firmness and spear diameter have been reported to be positively correlated with spear quality and the number of marketable spears, respectively (García et al., 2022). In asparagus, earliness refers to faster spear emergence and ensures early production (that is, the production of marketable shoots during the first 2 weeks of harvesting; Ellison et al., 1960), and is consequently another pivotal target of current breeding programs. Beyond spear-related quality traits, improvement of vigor in parental lines is essential to increase hybrid seed and crown production for commercialization. Improved parental lines showing asparagus foliage with more stems and phylloclades and larger spear size are highly desirable, as positive correlations have been found between this latter trait and hybrid seed production and yield (Anido and Cointry, 2008). Consequently, phylloclade density, number of stems, and stem length have been considered to be proxy traits of asparagus vigor in breeding programs (Kole et al., 2015). Similarly, hybrid seed production requires flowering synchronization between parental lines in order to schedule crosses, highlighting the importance of seeking variability in flowering time in male and female asparagus plants. Despite their importance in sustaining asparagus breeding, systematic analyses of the phenotypic diversity of these traits in breeding materials and related wild species have lagged behind, hampering the full exploitation of asparagus germplasm.

Several species related to garden asparagus exhibit different ploidy levels: in particular, tetraploid (4x) and hexaploid (6x) genotypes of *Asparagus maritimus* have been observed in plants selected from wild populations. Similarly, variation in ploidy has been observed in wild populations of *A. officinalis*, where 4x, 8x, and 10x individuals have been reported (Mousavizadeh et al., 2016). Diploid cultivars of *A. officinalis* represent a large fraction of worldwide asparagus production, although ‘Violetto d’Albenga’, ‘Morado de Huétor’, ‘Cereseto’, and ‘Poire’ are tetraploid genotypes of asparagus cultivated in Italy, Spain, and South America (Falavigna and Fantino, 1985; Moreno et al., 2006; Garcia et al., 2021). To date, few studies have been carried out to unravel the genetic diversity of cultivated diploid asparagus and its polyploid relatives. Analysis of an asparagus panel of 173 doubled haploids, five wild relatives, and three tetraploid interspecific lines carried out using amplified fragment length polymorphisms (AFLPs) has shown that these latter groups account for the largest fraction of genetic diversity observed in asparagus, while cultivated asparagus genotypes show a narrow genetic basis (Riccardi et al., 2011). Similarly, an analysis of genetic diversity carried out using sequence-related amplified polymorphism (SRAP) markers has corroborated the narrow genetic basis in the cultivated gene pool of garden asparagus (Amato et al., 2021). Other studies of molecular diversity carried out in doubled haploids and anther donors of breeding populations used and exploited in both Italian and international improvement programs have pointed out the lack of genetic variability in the germplasm of *A. officinalis*, as many hybrids on the market essentially originate from the same two progenitors, ‘Braunschweiger’ and ‘Argenteuil’, which are German and French lines, respectively (Mercati et al., 2015). The narrow genetic basis of cultivated asparagus germplasms is a contributing factor hampering the development of new hybrids; concurrently, there is a rising demand for the development of hybrids with higher yield and disease resistance, especially for cultivation in warm and wet environments (Garcia et al., 2021; Taguchi and Motoki, 2022). These concerns regarding genetic erosion in worldwide breeding populations are boosting the analysis of asparagus germplasm collections, as new sources of variability are pivotal to broaden current gene pool and for incorporating untapped diversity in future genetic improvement programs.

Species related to garden asparagus and their interspecific accessions are reservoirs of traits and genes that might help breeders to create new hybrids that can cope with climate change (Mousavizadeh et al., 2021). Recent studies on species related to garden asparagus have highlighted the importance of introducing new diversity into improvement programs to sustain pre-breeding (García et al., 2022). These valuable genetic resources have the potential to provide important traits such as tolerance to high levels of salinity and soil acidity, drought, and disease resistance (Garcia et al., 2021; Mousavizadeh et al., 2021).

In plant breeding and horticultural crops, target traits are often scored as ordinal categorical values instead of being measured on a continuous scale (Montesinos-López et al., 2015). Typically, plant disease resistance is scored on an ordinal categorical scale, but breeders often measure other continuous phenotypes by following the same approach: that is, by discretizing trait values into a small number of ordinal categories. Standard models for genome-wide association studies (GWASs) and genomic prediction (GP) assume a linear relationship between DNA markers and phenotypic values, constant variance, and that measured phenotype values are continuous and normally distributed (Montesinos-López et al., 2015). Consequently, GWAS and genome-enabled prediction of traits scored as ordinal categorical scales require appropriate models for analysis of these data.

In the present study, we assembled a panel of 378 asparagus genotypes, including plant materials currently used for breeding and exhibiting different ploidy levels, as well as diverse accessions sampled in their natural environments, and examined the diversity that characterizes the current gene pools of garden asparagus and related wild species. Leveraging field trials, we demonstrated the phenotypic diversity of this asparagus germplasm for nine traits relevant to spear quality and plant habitus. Moreover, we applied reduced-representation sequencing to characterize our asparagus collection in order to assess the molecular diversity and the extent of linkage disequilibrium (LD) in the diploid gene pool. Combining genotypic analyses and phenotypic information based on ordinal categorical values, we provide evidence that the collection assembled in the present study might be extensively used for GWAS and genome-enabled prediction to uncover the genetic bases of agronomically important traits and to accelerate asparagus breeding, respectively.

## Materials and methods

### Plant material

The asparagus panel assembled and examined in this study consisted of 378 genotypes, whose ploidy was determined by cytofluorometric analysis (data not shown). Of the 378 accessions included in the present panel, 80 were tetraploid, while the remaining genotypes were diploid (**Supplementary Table 1**). Among the 15 classes of genetic material included in the panel, the first three analyzed here were asparagus genotypes derived from anther cultures: specifically, 107 doubled haploids (DH) pure lines of *A. officinalis*, 28 lines (LI) obtained from *A. officinalis* anther cultures showing a high level of heterozygosity, and 1 DI-haploid (DI) individual with halved ploidy of tetraploid plants used as an anther donor (**Supplementary Table 1**). Along with the DH, LI, and DI classes, plants derived from interspecific crosses were included and examined in this panel: specifically, these were 37 interspecific lines (IL) obtained from crosses with other asparagus species (*A. maritimus* and *Asparagus acutifolius*) or of DI individuals with DH, LI, or hybrids of *A. officinalis*. In addition to the IL class, the panel included three interspecific individuals (IS) obtained from consecutive crosses between *A. officinalis*, *A. maritimus*, and *A. acutifolius* (i.e., the tetraploid interspecific hybrid plants named “OMAO”) (Riccardi et al., 2011); five plants derived from interspecific crosses (IC) between *A. officinalis* and *A. maritimus*; and 37 interspecific hybrids (IH), which were plants obtained by crossing *A. officinalis* genotypes belonging to the DH or LI groups with an interspecific line (**Supplementary Table 1**). Beyond these materials, the panel also included 26 heterozygous females (HF) and nine heterozygous males (HM) of *A. officinalis* collected in different natural environments (**Supplementary Table 1**). To assess the molecular diversity in species related to *A. officinalis*, 18 A*. maritimus* plants obtained by mass selection (MS) and 18 A. maritimus wild plants collected on the coast of northern Italy (MW) were included (**Supplementary Table 1**). Finally, the panel also included 18 commercial hybrids (CH), 25 experimental hybrids (EH) obtained during the course of more than 40 years of genetic improvement, three *A. officinalis* hermaphroditic individuals (HE), and 43 backcrosses (BC) obtained by crossing an experimental hybrid producing entirely green shoots with its female parent as part of a breeding program aiming to develop “all-green” asparagus hybrids (**Supplementary Table 1**). A subset of the doubled haploids and interspecific lines examined in this study had been previously characterized in other studies using AFLP markers (Riccardi et al., 2011).

### Phenotyping of plant material

During the 2021 growing season, a subset of 131 of the 378 genotypes included in the asparagus panel were phenotyped for nine traits at the CREA-GB experimental field in Montanaso Lombardo (Lodi, Italy, latitude 45.34, longitude 9.45) (**Supplementary Table 2**). Each genotype was arranged in plots consisting of a variable number of plants (from two to five, with a few exceptions consisting of a single plant), with no replicates (**Supplementary Table 2**). The following spear quality traits were considered: spear emergence, intensity of anthocyanic coloration in the spear, spear diameter, spear head firmness, and opening of bracts. In addition, traits linked to plant vigor and seed production were scored. These traits were phylloclade density, number of stems, stem length, and flowering time. The sex of the plants was recorded by looking for the presence/absence of stamens in open flowers to identify male/female plants, respectively (**Supplementary Table 1**). All these traits were measured under field conditions using the score values and following the criteria reported in **Supplementary Table 3**, which are briefly described below.

Spear emergence was scored as the number of spears that reached a specific phenological stage (height of 20 cm) on the same day. ‘Erasmus’ hybrid and plants belonging to the ‘Violetto d’Albenga’ population are considered early, producing approximately 60% 20-cm spears at the beginning of the season, while genotypes considered late produce fewer and shorter shoots (**Supplementary Table 3**).

Regarding the intensity of anthocyanic coloration in spears, a large fraction of plant material shows intermediate values that are attributed to the combination of green spears with purple coloration in the tip, bracts, and basal part. However, entirely green asparagus spears (such as the hybrid ‘Bacchus’) were classified with a lower score, while plants of the variety ‘Violetto d’Albenga’, which are characterized by completely purple spears, were used as the benchmark for the highest score (**Supplementary Table 3**).

For spear diameter, a low value was assigned to plants producing stems smaller than 10 mm, and higher values were assigned to plants having a caliber greater than 20 mm (**Supplementary Table 3**). This trait can be measured directly on the spears, or (as in this study, in which no spears were collected) can be deduced by assessing the caliber of the stems.

Spear head firmness was assessed by observing the shape of the shoot apex and attributing lower values to tips with enlarged or not very closed bracts and higher values to asparagus plants with very closed bracts at the tips (such as plants belonging to the ‘Violetto d’Albenga’ population), resembling the shape of a very well-sharpened pencil. The opening of bracts was observed both at the level of spears and that of the plant’s stems; lower values were attributed to plants with closed central bracts (indicating higher spear quality) and higher values to stems that tended to blossom quickly and exhibited very open bracts that were not particularly close to the stem (**Supplementary Table 3**).

Phylloclade density was measured by assessing several features: type of cladophylls (long or short), position (clumped or single), and distribution on the branch. This trait indirectly reflects the photosynthetic potential of plants. In the present study, lower scores were given to plants characterized by few thin phylloclades, while the highest score was given in the case of phylloclades evenly distributed on the branch (**Supplementary Table 3**).

Fern analysis was carried out according to established protocols (Anido et al., 1997), analyzing the number, diameter, and length of stems. Plants with less than three stems were assigned to lower classes, while plants with more than six stems emerging from the rhizome were assigned to higher classes (**Supplementary Table 3**).

For stem length, the lowest values were given to shorter plants observed in the field, while taller plants (such as OMAO) obtained the highest stem length values (**Supplementary Table 3**).

Finally, flowering time was measured in terms of the phenological status of the flowers of each accession on the day of the survey. Early plants showing mostly open flowers were assigned to lower classes, while late plants showing multiple buds and closed flowers were assigned to higher classes (**Supplementary Table 3**).

### DNA extraction and genotyping

DNA was extracted from leaf tissues collected from young plants at the beginning of the vegetative season using the GenElute™ Plant Genomic DNA Miniprep Kit (Sigma-Aldrich, St. Louis, MO, USA) following the manufacturer’s protocol. Quality control was carried out to assess the integrity of genomic DNA using the TapeStation system (Agilent, Santa Clara, CA, USA) with Genomic ScreenTape. DNA quality and concentration were assessed using a NanoDrop spectrophotometer and the dsDNA assay kit (Qubit BR; Life Technologies, Carlsbad, CA, USA), respectively.

Genotyping of DNA samples was carried out using double digest restriction-site associated DNA (ddRAD) sequencing at the Functional Genomics Laboratory (University of Verona, Italy) following published protocols (Peterson et al., 2012), with the following modifications. For each sample, 200 ng of gDNA was digested for 2 h at 37°C, followed by 20 min at 65°C and 2 h at 75°C with 2 U of *Ape*KI and 2 U of *Mse*I (New England Biolabs, NEB, Ipswich, MA, USA) in 1× NEB 3.1 buffer, for a final volume of 20 μl. Digestion products were verified by running the digested DNA and the intact genomic DNA on a 4150 TapeStation using the Genomic DNA assay (Agilent Technologies). Digested DNA was ligated to a double-stranded barcoded adaptor (previously annealed, 0.025 μM final concentration) and a double-stranded common adapter Y bringing a biotin tail (previously annealed, 0.025 μM final concentration) with 1 U of T4 DNA ligase (Invitrogen, Carlsbad, CA, USA) in the presence of 1× ligase buffer at a final volume of 50 μl. A total of 24 different barcoded adaptors were employed to uniquely identify 24 samples at a time. The ligation reaction was performed in a thermocycler for 10 min at 30°C and 4 h at 22°C, followed by inactivation for 30 min at 65°C. Samples were subsequently pooled at equimolar concentration and purified using beads (0.75× volume of AMPure XP; Beckman Coulter, Brea, CA, USA) following the manufacturer’s instructions. The purified pool was resuspended in 25 μl of water. Next, 24 µl of biotinylated DNA fragments was captured with 1× volume of Dynabeads M-270 streptavidin (Invitrogen), according to the manufacturer’s instructions. Beads with the captured DNA were resuspended in 60 µl of water. Half of the pool (30 μl) was subsequently amplified in a 50-μl reaction volume using 2 U of Taq Phusion polymerase in the presence of 1× Taq Phusion HF buffer, 0.3 mM of dNTPs, and two different primers—Primer PCR1 (0.5 μM) and PPIX Illumina Index (0.5 μM)—the latter including an index for Illumina sequencing. A total of 16 PPI Illumina Index primers with 16 different Illumina indexes were used, allowing multiplexing of 16 pools (=378 samples) at a time. Amplification was performed according to the following thermal PCR program: 30 s at 98°C; 15 cycles of 10 s at 98°C, 30 s at 65°C, and 30 s at 72°C; and 5 min at 72°C for final elongation. Final ddRAD libraries were purified with beads (0.65× AMPure XP; Beckman Coulter), and their size distribution was determined on a 4150 TapeStation using a D1000 HS Assay (average insert size was ~560 bp). ddRAD libraries were finally quantified *via* qPCR, pooled at equimolar concentration, and sequenced on an Illumina NovaSeq 6000 platform using a 2 × 150 bp sequencing protocol.

### SNP calling procedure

To enable an unbiased diversity analysis of different diploid and polyploid species within the *Asparagus* genus, ddRAD sequencing data were analyzed using a reference-free pipeline implemented in Stacks 2.62 (Rochette et al., 2019). Firstly, raw reads obtained after ddRAD sequencing were processed using the Stacks 2.62 built-in program “process_radtags” to filter and demultiplex sample reads using default parameters. Loci were built following the *de novo* pipeline in Stack 2.62, analyzing forward reads with “ustacks” to identify read tags and shape loci and for single-nucleotide polymorphism (SNP) calling within individuals. Subsequently, “cstacks” was used to create a comprehensive catalog of all loci identified with “ustacks”, and “sstacks” was used to identify sample loci matching with the catalog using default parameters. Following the *de novo* analysis procedure, the “tsv2bam” and “gstacks” programs were used to incorporate paired-end (PE) reads in loci and to assemble a contig for each locus and call SNPs.

To obtain information regarding the genomic coordinates of loci included in the catalog, *de novo* analysis was integrated with the reference genome for asparagus. Loci included in the catalog created with “gstacks” using *de novo* assembly were mapped to the reference sequence of *A. officinalis* downloaded from the Phytozome website (https://phytozome-next.jgi.doe.gov/info/Aofficinalis_V1_1), mapping the catalogs of tags using bwa v0.7.17 (Li and Durbin, 2009). Using the stacks-integrate-alignments python 3 script included in Stacks 2.62, the genomic coordinates of SNPs mapped to the catalogs were lifted over to the reference sequence for asparagus using a minimum alignment coverage (–min_aln_cov) of 0.8 and considering loci mapping with a percentage identity (–min_pct_id) higher than 0.8. This subset of SNPs identified using *de novo* analysis and integrated within the reference sequence for asparagus was used to compute LD analysis, GWAS, and genome-enabled prediction models. Missing data obtained from the aforementioned SNP calling procedure were imputed using Beagle v4.1 (Browning and Browning, 2016) with default parameters.

### Diversity and genome-wide association analyses

Principal component analysis (PCA) was carried out using the full set of SNPs identified by applying the reference-free pipeline of Stacks 2.62 to ddRAD sequencing using base functions implemented in R (R Core Team, 2020), without applying either pruning or clumping procedures. PCA plots were created in R using the ggplot2 package (Wickham, 2016).

Pairwise LD was measured using SNPs of diploid asparagus genotypes integrated into the reference sequence, with *r* ^2^ values (Hill and Robertson, 1968) computed using vcftools (Danecek et al., 2011) with the “–geno-r2” option. Before computing *r* ^2^ values, SNPs were thinned using a window of 50,000 bp, and they were subsequently used to compute pairwise *r* ^2^ values. The resulting list of pairwise values was imported into R and used to plot the decay of LD as a function of SNP site distance across the 10 asparagus chromosomes.

For both binary and ordinal categorical traits, GWAS was carried out using a proportional odds logistic mixed model implemented in the POLMM R package (version 0.2.3) (Bi et al., 2021). In order to conduct GWAS using POLMM, the full set of SNPs integrated into the reference sequence for asparagus was tested, including sex and the top 10 principal components as covariates to account for sex dimorphism and population stratification, respectively. For sex, GWAS was carried out using POLMM with the full set of SNPs and the top 10 principal components as covariates. GWAS was carried out in two stages following the POLMM manual. In the first step, a dense genomic relationship matrix was computed using all available SNPs, and null models including fixed covariates (top 10 principal components and sex) were fitted using the POLMM R package. Subsequently, score tests were performed on each SNP using the saddle-point approximation method to compute *p*-values. We considered all variants with a *p*-value lower than 1*10^–6^ to be genome-wide significant. Manhattan plots of GWAS results were generated using the qqman package and R version 4.2.1 (Turner, 2018). GWAS analysis was complemented by the identification of candidate genes in the flanking regions of significant markers. Using the Plant Epigenome Browser tool available at https://epigenome.genetics.uga.edu/PlantEpigenome/, a portion of approximately 60 kb around the position of the markers found to be associated with sex and with anthocyanic coloration of the spears was analyzed to identify genes annotated in the aforementioned portions of the corresponding chromosomes. Once the FASTA sequences of the genes under investigation had been downloaded, BLASTn alignments were carried out on the NCBI website (National Centre for Biotechnology Information, https://blast.ncbi.nlm.nih.gov/Blast.cgi) to find homologous genes.

### Genome-enabled prediction of ordinal categorical traits

In the present study, leveraging phenotypic information and SNPs detected with ddRAD sequencing data, threshold genomic best linear unbiased predictor (TGBLUP) models (Montesinos-López et al., 2015) were used to implement genome-enabled prediction in order to assess whether our collection might be used to accelerate asparagus breeding. These models have been discussed extensively elsewhere (Montesinos-López et al., 2015), so we will only briefly introduce the TGBLUP models implemented in the present study and the Brier Score (BS) used to estimate the predictive ability of these models.

The ordinal response variables *y_jk_* used to implement a TGBLUP module, where *j* indicates the genotype and *k* represents sex, can assume ordinal values from C=1 to C=9, following a multinomial distribution:


$$\left(y_{jk\left(C=1\right)},\ldots ,\;y_{jk\left(C=9\right)}\right)∼\;Multinomial\;\left(N_{jk\;},\;\pi_{jk\left(C=1\right)},\;\ldots ,\;\pi_{jk\left(C=9\right)}\right),$$

where *N_jk_* represents the number of observations and $\pi_{jk\left(C=1\right)},\;\ldots ,\;\pi_{jk\left(C=9\right)}$ are the probability values from C=1 to C=9 for the *j*th line and *k*th sex. TGBLUP models assume that the response variable *y_jk_* is generated from a normally distributed latent “liability” variable *l_jk_* (Sorensen et al., 1995; Montesinos-López et al., 2015) that generates the nine observed categories, as follows:


$$l_{jk}=X_{jk}^{T}\beta +Z_{jk}^{T}u+\;e_{jk},$$

where *e_jk_* is the error term, normally distributed with mean 0 and standard deviation equal to 1 (that is, $e_{jk}\;\sim N\left(0,1\right)$ ), while $X_{jk}^{T}\beta$ and $Z_{jk}^{T}u$ are the linear predictors.

For each ordinal response variable $l_{jk}$ , 8 (C–1 = 8 where C=9) unknown γ thresholds must be estimated as $\gamma_{min}<\gamma_{1}<\ldots <\gamma_{8}<\gamma_{max}$ with $\gamma_{min}=-\infty$ and $\gamma_{min}=\infty$ , using the following conditions:


$$y_{jk}=\;\{\begin{array}{c} 1\;if\;-\infty <l_{jk}<\gamma_{1}\;\\ 2\;if\;\;\;\gamma_{1}<l_{jk}<\gamma_{2}\\ \ldots \\ 8\;if\;\;\;\gamma_{7}<l_{jk}<\;\infty \end{array}.$$

In TGBLUP models, the link function is the cumulative probit Φ(.) , i.e., the cumulative distribution function of a standard normal distribution, known as probit link, while ${\text{Φ}}^{-1}$ represents the inverse function. The link function used in our models has been described and discussed extensively elsewhere (Montesinos-López et al., 2015).

The latent “liability” variable *l_jk_* underlying TGBLUP models has been described previously in Montesinos-López et al. (2015) in reference to “Model 2”. Specifically, this “liability” variable is modeled using the following sets of linear predictors:


$$l_{jk}=\;S_{k}+g_{j}+\epsilon_{jk},$$

where $S_{k}$ represents the fixed effect of *k*th sex; $g_{j}$ represents the additive genetic value of the *j*th line, normally distributed with mean 0, as $g_{\;}∼N\left(0,G\sigma_{g}^{2}\right)$ , where $\sigma_{g}^{2}\;$ represents the additive genetic variance and **G** is the genomic relationship matrix (VanRaden, 2008); and $\epsilon_{jk}$ is the error term for the *j*th line having the *k*th sex, which is normally distributed with mean 0 and standard deviation equal to 1, as follows: $\epsilon_{jk}\;∼N\left(0,1\right)$ . The additive genetic value was computed using the following formula:


$$g_{j}=\sum_{n=1}^{p}x_{jn}b_{n}$$

where $x_{jn}$ is the genotype of the *j*th line at marker n and $b_{n}$ is the corresponding effect of marker n .

Finally, the predictive ability of the TGBLUP models was estimated by computing the BS (Brier, 1950; Montesinos-López et al., 2015; Montesinos-López et al., 2020) using a leave-one-out (LOO) cross-validation strategy, as follows:


$$BS\;=\left(n^{-1}\sum_{i=1}^{n}\;\sum_{c=1}^{g}\;\left({\hat{\pi }}_{ic}-d_{ic}\right){\;}^{2}\;\right)/2,$$

where $\left({\hat{\pi }}_{ic}-d_{ic}\right){\;}^{2}$ is the average square difference between ${\hat{\pi }}_{ic}$ predictions and $d_{ic}$ classes for observation *i* classified into category *c*. Lower values of BS indicate higher predictive ability of the model, while higher values of BS indicate lower predictive ability. Threshold models were fitted using the BGLR package (Pérez and de los Campos, 2014) implemented in R (R Core Team, 2020), with previously published modifying scripts (Montesinos-López et al., 2015).

## Results

### Analyses of genetic and phenotypic diversity

To examine the genetic diversity in breeding populations of garden asparagus and other asparagus species, ddRAD sequencing was applied to fingerprint a panel of 378 genotypes; this panel encompassed several groups of related and unrelated individuals, including doubled haploids of *A. officinalis*, *A. maritimus* genotypes, and interspecific crosses with *A. officinalis* (**Supplementary Table 1**). As our collection included both diploid and polyploid genotypes, ddRAD sequencing data were analyzed using a reference-free pipeline or *de novo* analysis to obtain an unbiased estimate of genetic diversity. Analysis of the subset of diploid asparagus genotypes showed that, on average, approximately 2.59 M high-quality PE reads per sample were obtained, targeting, at 3× coverage, approximately 2.3% of the asparagus reference sequence (Harkess et al., 2017). Overall, ddRAD sequencing enabled the identification of 266,062 polymorphic SNPs, 254,989 of which were subsequently anchored in the 10 asparagus pseudomolecules for genome-wide association analyses. The largest number of SNPs (more than 30,000) was detected on chromosomes 1 and 7, while the lowest number of SNPs (approximately 10,000) was detected on chromosomes 2, 6, 9, and 10 (**Supplementary Figure 1**).

**Figure 1:**
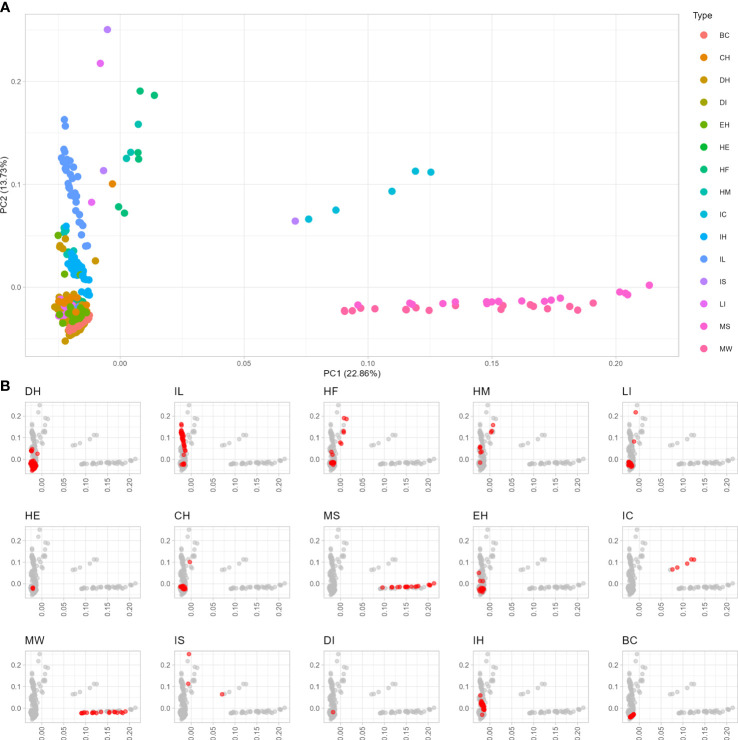
Diversity analysis of 15 groups of different asparagus genotypes. **(A)** Principal component analysis of the entire collection of 378 asparagus genotypes examined in this study. **(B)** Principal component plots in which different classes of asparagus examined in this study are highlighted in red. BC, back-cross; CH, commercial hybrid; DH, double haploid; DI, dihaploid; EH, experimental hybrid; HE, hermaphrodite; HF, heterozygous female; HM, heterozygous male; IC, interspecific cross; IH, interspecific hybrid; IL, interspecific line; IS, interspecific individual; LI, line obtained by anther culture; MS, maritimus selected; MW, maritimus wild-type.

To examine the diversity and population stratification of the asparagus panel, the resulting SNPs were subjected to PCA, which showed that the first and second components explained 22.86% and 13.73% of the total genetic variability, respectively (**Figure 1**). The genetic diversity space, as revealed by PCA carried out on ddRAD data, overall showed several genetic clusters that correlated with the 15 classes of asparagus plants examined in this study. The first main cluster of genotypes included both *A. maritimus* accessions sampled in their natural environment (MW) or subjected to mass selection (MS) (**Figure 1**). Similarly, genotypes derived from enforced crosses of *A. officinalis* with *A. maritimus* and *A. acutifolius* (IS) and interspecific crosses of *A. officinalis* with *A. maritimus* (IC) formed a second group of asparagus plants (**Figure 1**). The third largest cluster indicated by PCA included doubled haploid lines of *A. officinalis* (DH), interspecific lines (IL) derived from *A. officinalis* and DI-haploid lines (DI), one hermaphrodite genotype of *A. officinalis* (HE), commercial (CH) and experimental (EH) hybrids and backcrosses (BC) of *A. officinalis*, and interspecific hybrids (IH; **Figure 1**). Heterozygous male (HM) and female (HF) plants of *A. officinalis*, which are not derived from anther cultures, formed a close genetic cluster along with some IS and CH genotypes and lines showing high levels of heterozygosity (LI; **Figure 1**). Overall, the analysis of molecular diversity showed a narrow genetic basis for *A. officinalis* genotypes included in current breeding populations (e.g., the DH group; **Figure 1**) and indicated that genotypes included in the IL, MW, and MS groups might be a source of untapped diversity that could be used to sustain asparagus breeding.

**Figure 2:**
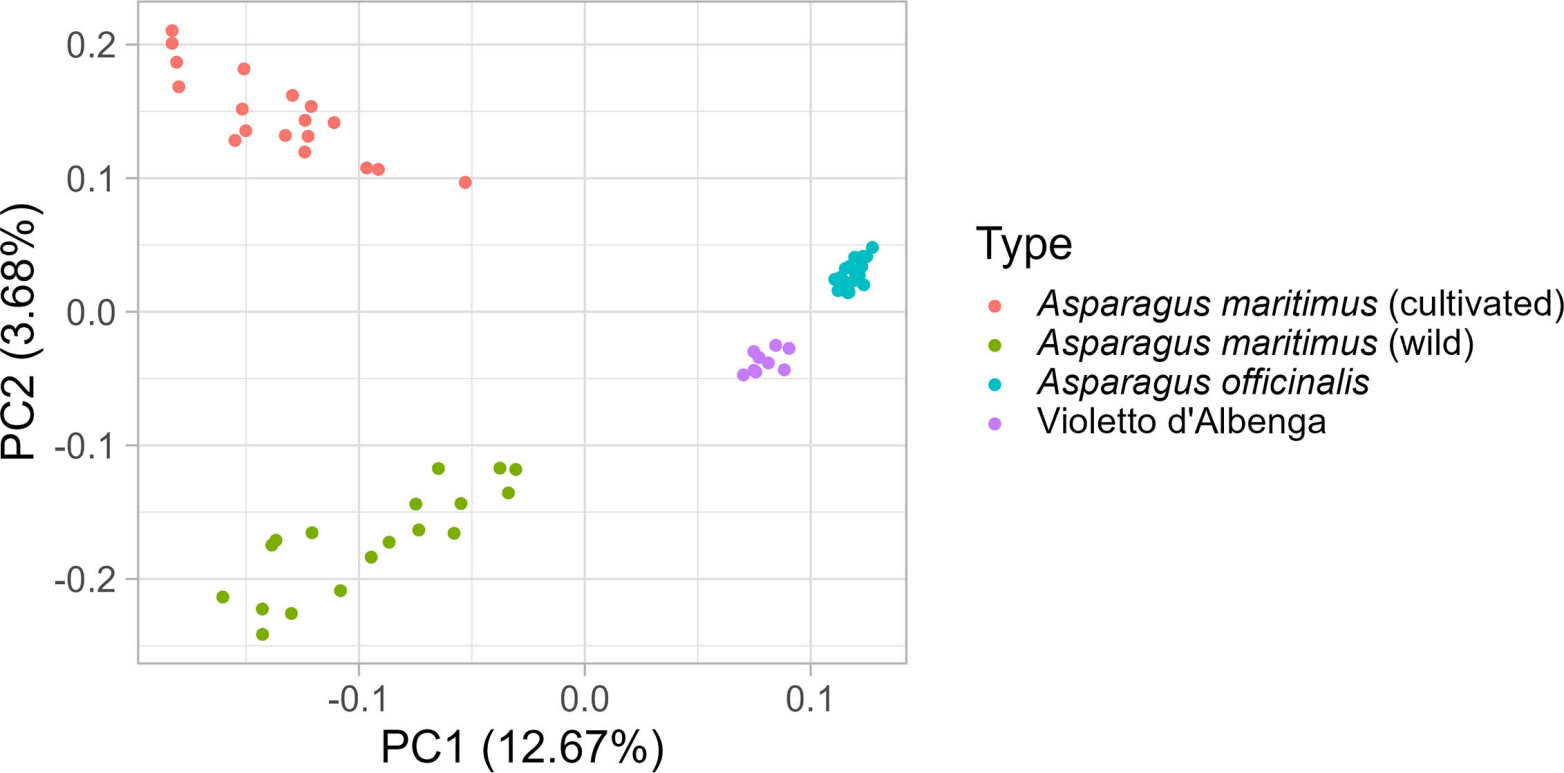
Diversity analysis of *Asparagus maritimus*, *Asparagus officinalis*, and ‘*Violetto d’Albenga*’ genotypes. Principal component analysis carried out on a reduced set of 74 asparagus genotypes, including plants belonging to the ‘Violetto d’Albenga’ population along with accessions of *A. maritimus* and *A. officinalis*.

To shed light on the origin of the ‘Violetto d’Albenga’ population, PCA was carried out on a reduced set of 74 individuals, including 36 wild and cultivated genotypes of *A. maritimus*, 29 diploid and tetraploid genotypes of *A. officinalis*, and nine genotypes of ‘Violetto d’Albenga’ (**Supplementary Table 4**). This analysis highlights the signatures of genetic differentiation between cultivated and wild genotypes of *A. maritimus*, indicating that mass selection carried out on this species has contributed to the alteration of allele frequencies in cultivated genotypes of this species (**Figure 2**). Interestingly, individuals belonging to the ‘Violetto d’Albenga’ population formed a distinct genetic cluster that was closer to the group of *A. officinalis*, corroborating the hypothesis that the ‘Violetto d’Albenga’ population might have been generated from interspecific crosses of garden asparagus with *A. maritimus* or others wild asparagus present in the original growing areas of ‘Violetto d’Albenga’ (**Figure 2**).

**Figure 3:**
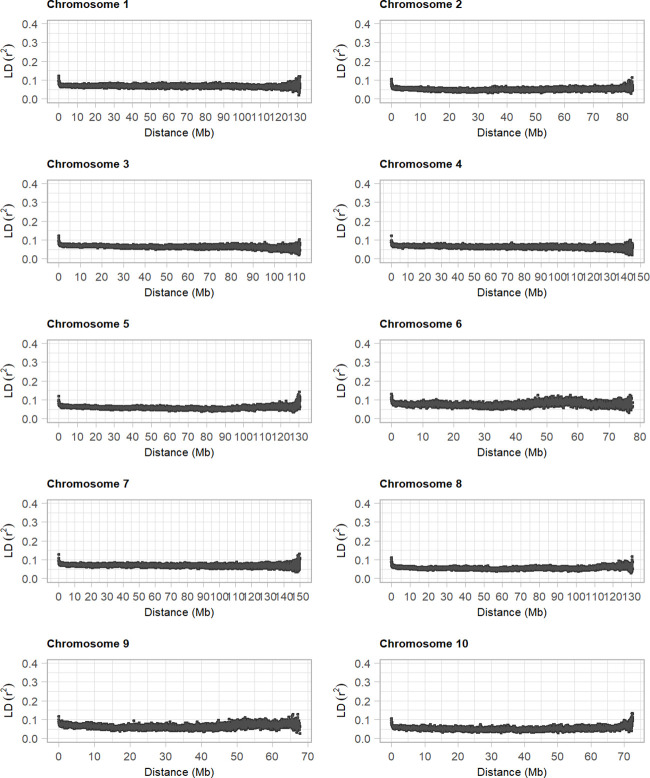
Decay of linkage disequilibrium in diploid asparagus genotypes. The 10 plots show the average *r^2^* values computed between pairs of loci in 100-kb bins across the asparagus chromosomes.

Polymorphic SNPs detected in the panel of diploid asparagus and integrated into the reference sequence were filtered using a minimum allele frequency higher than 0.05. The resulting markers were used to estimate the mean LD for each chromosome in 10-kb bins using *r* ^2^. This analysis showed that the average LD estimates started from *r* ^2^ = 0.15 for bin distances<100 kb and declined very rapidly to *r* ^2^ values of 0.05 with increasing bin distance, and that all 10 asparagus chromosomes exhibited the same pattern of mean LD decay (**Figure 3**). Overall, our analysis, which was carried out in a diverse panel including related individuals (e.g., the BC group) and plants with unknown pedigree, indicated a rapid decay of LD in diploid asparagus.

**Figure 4:**
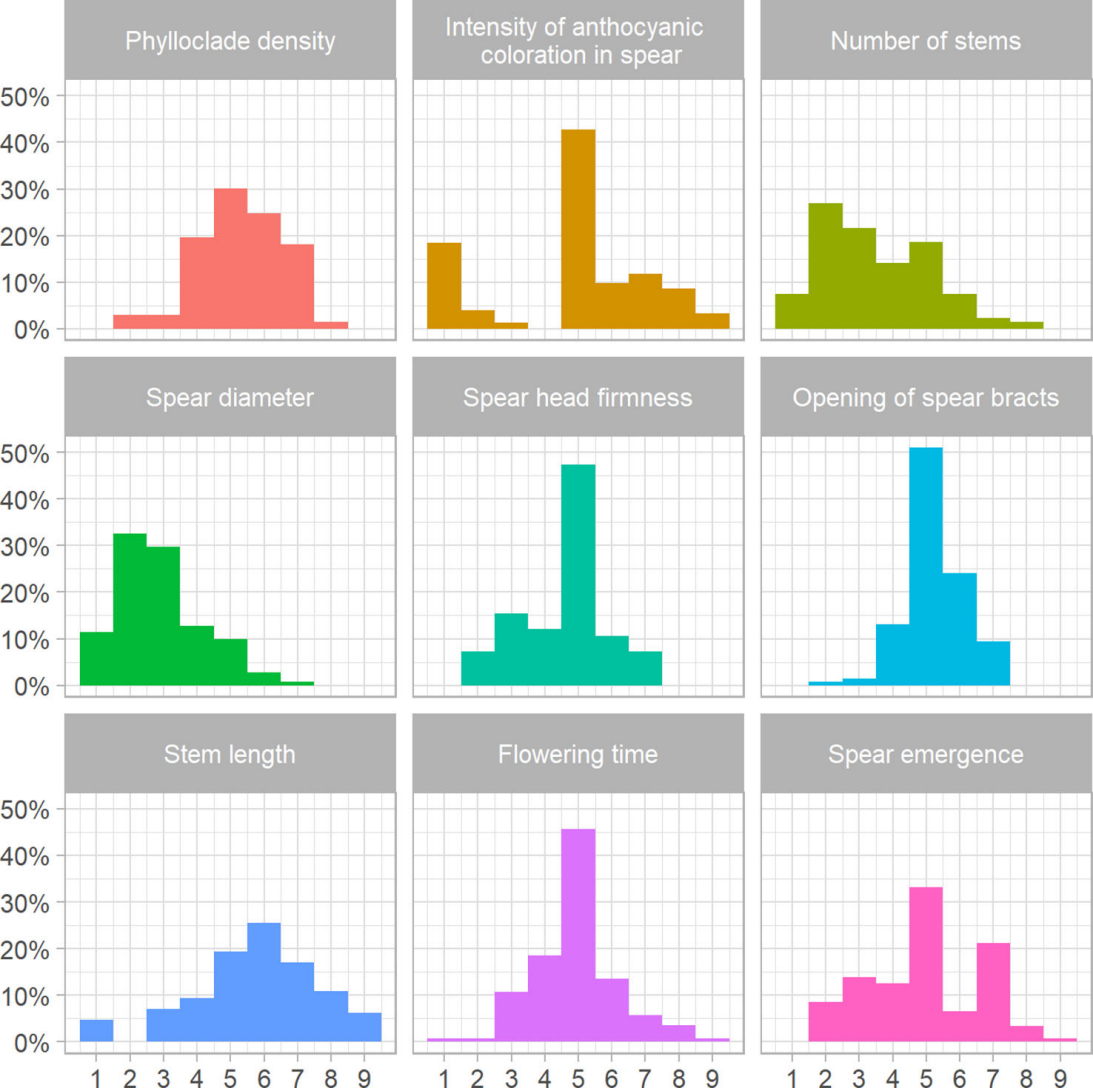
Frequency distributions of scores measured for quality and seed production traits. Histograms show the frequency distribution of nine agronomic traits, each measured on an ordinal categorical scale from 1 to 9, in 131 genotypes of asparagus.

To complement the genetic diversity analyses, a subset of 131 of the 378 individuals examined using ddRAD sequencing was phenotyped for nine traits relevant to spear quality and seed production, using an ordinal categorical scale from 1 to 9 (**Supplementary Table 2**). Analysis of the frequency distribution for spear emergence showed that approximately 30% and 20% of the analyzed material fell in the fifth and seventh score classes, respectively (**Figure 4**). Only three asparagus genotypes were very early and were assigned to the highest score class, while 10 accessions (approximately 6.5% of the total) showed late emergence and were classified in the second class. Our phenotypic analysis did not allocate any plants to the first class (**Figure 4**). Analysis of the intensity of anthocyanic coloration in spears showed that more than 40% of the asparagus genotypes fell into the fifth scoring class (**Figure 4**). These shoots showed green coloration along the entire surface with the presence of a pinkish ring at the base of the shoot and anthocyanic coloration of the bracts. Approximately 20% of the asparagus genotypes examined were allocated a score of 1; these plants showed completely green shoots without any visible trace of anthocyanic coloration, and this peculiar pattern was maintained even at the level of the entire vegetative part of the plant, even under particularly cold environmental conditions (**Figure 4**; **Supplementary Tables 2**, **3**). The five accessions falling into the scoring class “9” showed completely purple spears that tended to become discolored during complete plant development and become dark green stems; these are typical of the ‘Violetto d’Albenga’ population (**Figure 4**; **Supplementary Tables 2**, **3**). The frequency distributions for spear diameter showed that approximately 10% of the plants fell in the lowest-scoring class (**Figure 4**). Phenotypic analysis of spear head firmness showed that approximately 50% of the vegetative material received intermediate scores (scoring class 5), with approximately 5% of the accessions producing shoots showing very tight and closed tip conformation (**Figure 4**; **Supplementary Table 3**). The analysis of the opening of bracts was carried out at the level of the spears and showed a frequency distribution similar to that observed for spear head firmness. For this trait, asparagus genotypes with a more closed bract conformation, which are most valued by consumers, were those falling in the two lowest scoring classes, which represented approximately 2% of the genotypes examined in the collection (**Figure 4**; **Supplementary Table 3**). The frequency distribution for phylloclade density showed that most plants had intermediate phenotypes falling into classes 4, 5, 6, and 7, while approximately 1% and 1.3% of plants showed sparse cladophylls (class 1) and dense phylloclades (class 7), respectively. Moreover, our analysis showed that no plants were assigned to the higher scores (class 9) (**Figure 4**; **Supplementary Tables 2**, **3**). Analysis of the frequency distribution for number of stems showed that approximately 10% of the plants fell into the lowest-scoring class, indicating that the examined panel contained plants with a small number of stems (**Figure 4**). The frequency distribution for stem length showed that intermediate heights (scoring classes 5 and 6) were the most widespread, while approximately 4% and 5% of asparagus genotypes showed short and very tall stems, respectively (**Figure 4**). Finally, analysis of flowering time showed a similar trend, with more than 40% of plants flowering at approximately the mid-season (middle of May); very few accessions were early- or very late-flowering (**Figure 4**). The same set of traits was analyzed separately in male and female plants, and in general, pronounced sexual dimorphism did not seem to be evident (**Supplementary Figure 2**).

**Figure 5:**
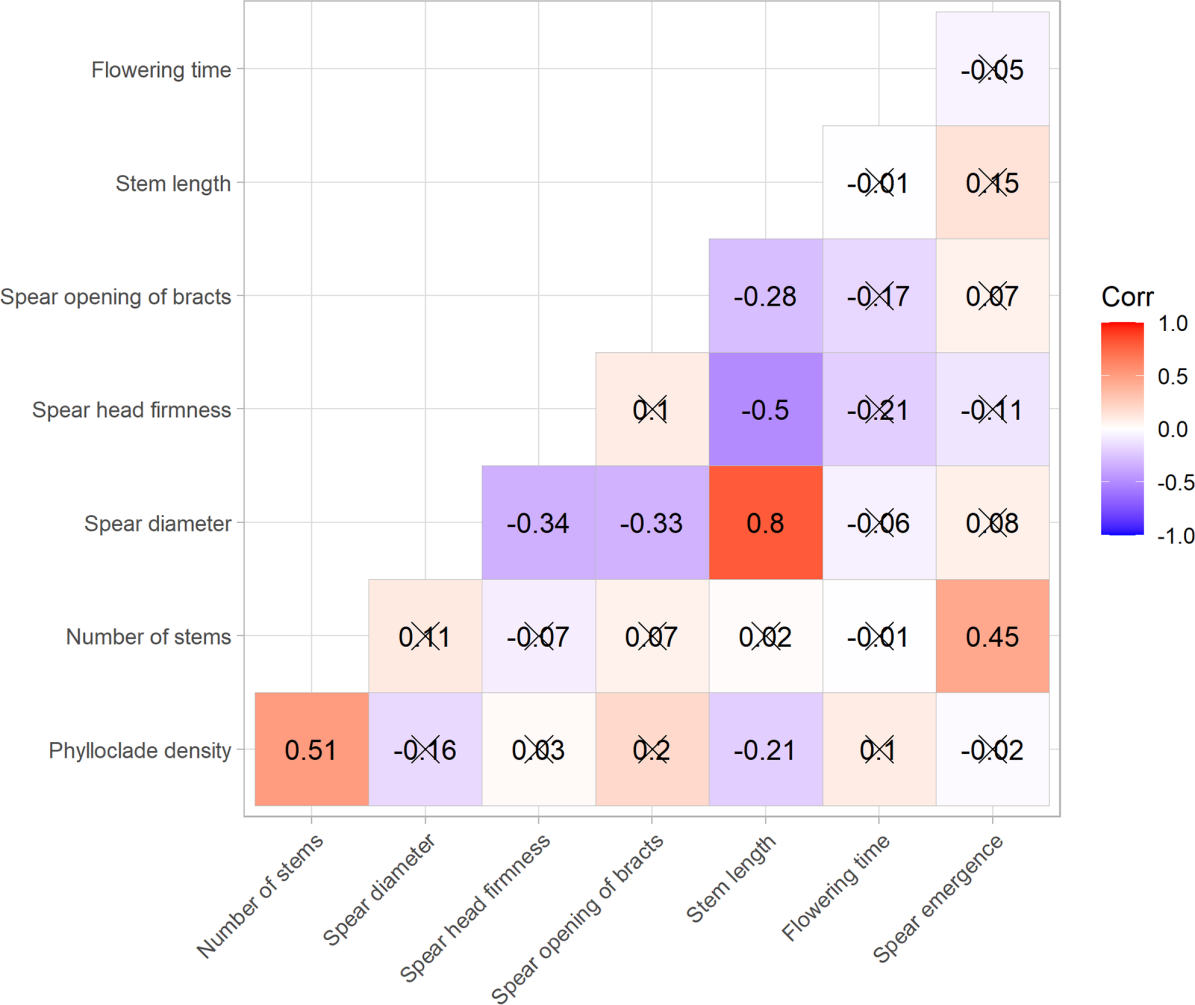
Heatmap of pairwise correlations computed among nine asparagus traits. Numbers reported within the heatmap represent polychoric pairwise coefficients between the ordinal categorical traits measured. Positive correlations are displayed in red and negative correlations in blue; correlations with associated *p*-values greater than 0.05 are marked with crosses.

To assess the relationships between the traits examined in this study, a pairwise correlation analysis was carried out using polychoric statistics (**Figure 5**). This analysis indicated the presence of positive and statistically significant correlations between spear diameter and stem length (r = 0.8, *p* < 0.05) and between density of phylloclades and number of stems (r = 0.51, *p* < 0.05) (**Figure 5**). Similarly, there was a significant positive correlation between number of stems and spear emergence (r = 0.45, *p* < 0.05) (**Figure 5**). Negative correlations with stem length were detected for density of phylloclades (r = −0.21, *p* = 0.04), spear head firmness (r = −0.5, *p* < 0.05), and opening of bracts (r = −0.28, *p* = 0.01). Similarly, spear diameter was significantly negatively correlated with spear head firmness (r −0.34, *p* < 0.05) and opening of bracts (r = −0.33, *p* < 0.05) (**Figure 5**).

### Genome-wide association analysis and genome-enabled prediction of sex and agronomic traits

**Figure 6:**
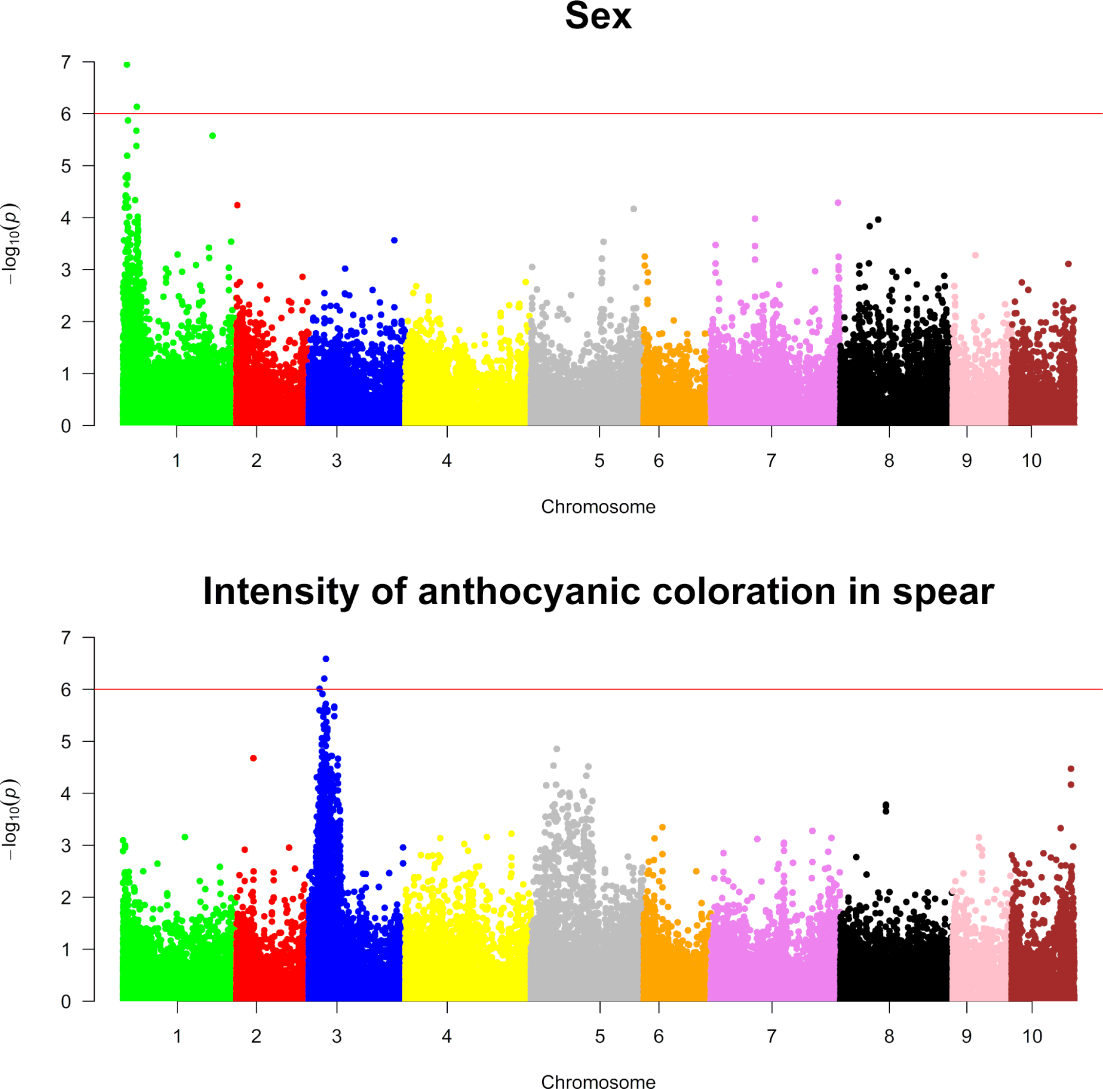
Genome-wide association analyses for sex and intensity of anthocyanic coloration in spears. The top and bottom panels show Manhattan plots of genome-wide association analyses carried out for sex and intensity of anthocyanic coloration in spears, respectively. The red line on each plot indicates the threshold for significance, which was fixed at $-{\text{log}}_{10}\left(p\;\text{value}\right)=6$ .

**Figure 7:**
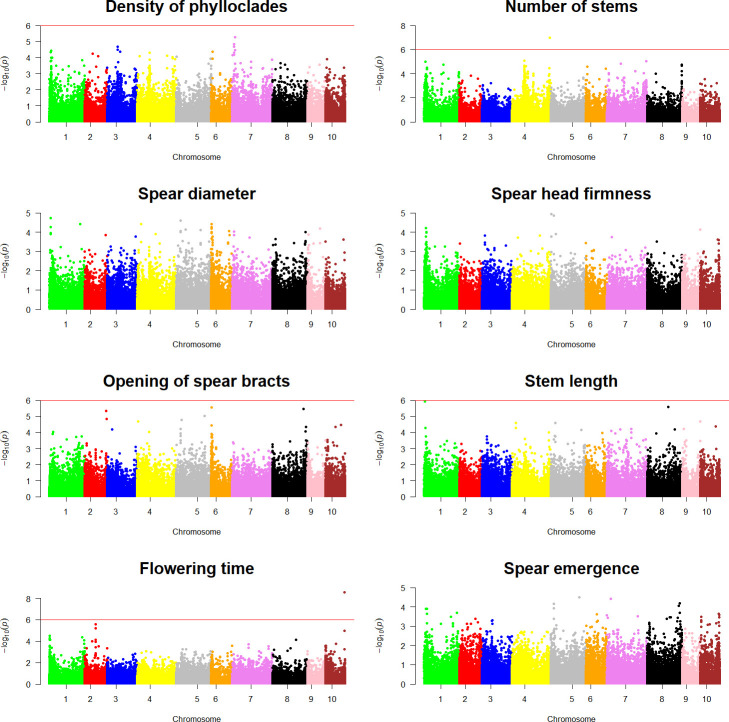
Genome-wide association analyses for asparagus quality traits. Each panel shows a Manhattan plot of a genome-wide association analysis carried out for a single trait (density of phylloclades, number of stems, spear diameter, spear head firmness, opening of spear bracts), stem length, flowering time, and spear emergence). The red line on each plot indicates the significance threshold of $-{\text{log}}_{10}\left(p\;\text{value}\right)=6$ .

Genotypic information and ordinal categorical phenotypic data were analyzed using a proportional odds logistic mixed model (POLMM) (Bi et al., 2021) in order to carry out GWAS for the nine agronomic traits investigated in this study and for sex. Using a significance threshold of $-{\text{log}}_{10}\left(p\;\text{value}\right)$ = 6, GWAS analyses detected markers statistically associated with sex determination and intensity of anthocyanic coloration in the spears in chromosome 1 and chromosome 3, respectively (**Figure 6**). Similarly, GWAS analyses for the remaining traits examined in this study detected statistically significant markers associated with number of stems and flowering time (**Figure 7**).

GWAS for sex determination identified two significant SNPs in the peak (SASPARAGUSV101_4691863 and SASPARAGUSV1_01_16384084) having *p*-values of $1.13*10^{-7}$ and $7.34*10^{-7}$ , respectively (**Supplementary Table 5**). Considering the low extent of LD (**Figure 3**), we decided to investigate the presence of annotated genes in a portion of the genome comprising approximately 30 kb upstream and 30 kb downstream of significant marker coordinates. Interestingly, SNP “SASPARAGUSV1_01_4691863” was mapped approximately 50 kb away from the putative boundary of the non-recombining region of chromosome 1/Y determining asparagus sex, corroborating the validity of the GWAS analyses carried out in this study (Harkess et al., 2017). Unlike the first marker, three genes were annotated in the region near the second marker (SASPARAGUSV1_01_16384084), coding for *caffeic acid 3-O-methyltransferase* or *nicotinate N-methyltransferase* (**Supplementary Table 7**).

For the intensity of anthocyanic coloration in the spear, two significant SNPs (SASPARAGUSV1_03_20222771 and SASPARAGUSV1_03_18321323) were detected in the peak: SASPARAGUSV1_03_20222771 showed a negative effect of 1.72, *p* = $2.70*10^{-7}$ , while SASPARAGUSV1_03_18321323 exhibited a similar effect, *p* = $6.85*10^{-7}$ (**Supplementary Table 6**). The genome annotation analysis indicated that there were five annotated genes close to the first marker (SASPARAGUSV1_03_20222771), while three were annotated around the second significant marker (SASPARAGUSV1_03_18321323) (**Supplementary Table 7**). Unlike the first two markers, the analysis of genes of the asparagus genome annotated within a 60-kbp window did not identify genes close to the third marker (SASPARAGUSV1_03_12831852). All the genes located in the above-mentioned portions of the genome either were not characterized or coded for transcription factors or enzymes; in some cases, the latter could be associated with the anthocyanin biosynthetic pathway, such as *AsparagusV1_03.907* and *AsparagusV1_03.908* (**Supplementary Table 7**).

A single significant SNP (SASPARAGUSV1_04_141920731), mapped in chromosome 4, formed the main peak of the association analysis for number of stems (**Figure 7**). This marker was associated with an effect of −2.67, *p* = $9.81*10^{-8}$ . This SNP was found to be closely linked to a gene coding for an ABC transporter C family member 5-like (*AsparagusV1_04.3451*) and another with an expansin-B3-like function (*AsparagusV1_04.3450*) (**Supplementary Table 7**).

Similarly, a single significant SNP (SASPARAGUSV1_10_72001201) formed the peak of the Manhattan plot for flowering time (**Figure 7**). This marker had a negative effect of 2.46, *p*- = $2.87*10^{-9}$ , and was found to be closely linked to two genes coding for an ABC transporter C family member 5-like (*AsparagusV1_10.1874* and *AsparagusV1_10.1875*). Two other genes with a subtilisin-like protease function (*AsparagusV1_10.1872* and *AsparagusV1_10.1873*) and a gene with a myb-related protein 305-like function (*AsparagusV1_10.1876*) (**Supplementary Table 7**) were also closely linked to SASPARAGUSV1_10_72001201 SNP.

**Figure 8:**
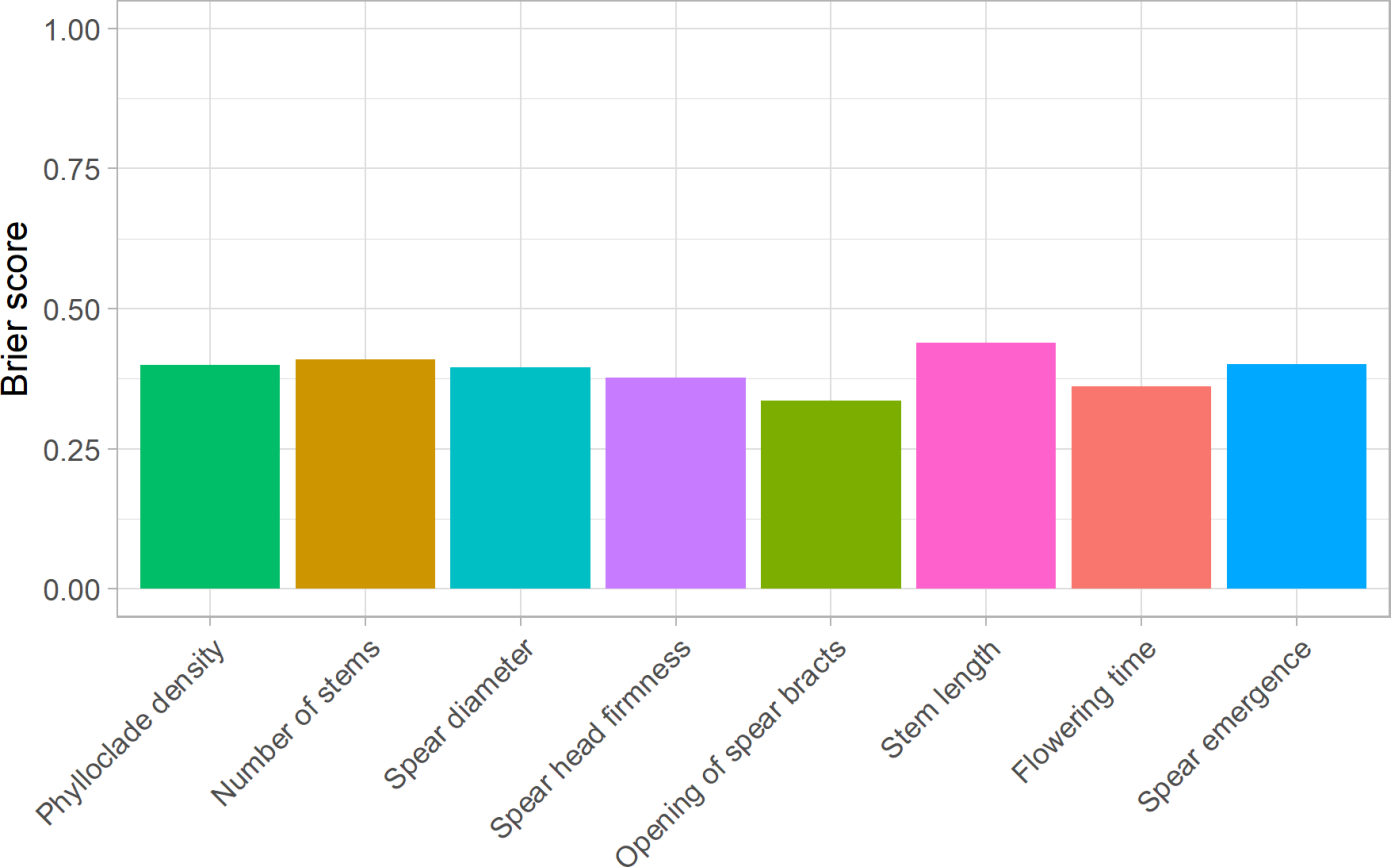
Predictive ability of genome-enabled prediction models for asparagus quality traits. Each bar shows Brier Scores obtained using leave-one-out cross-validations carried out for a single trait (phylloclade density, number of stems, spear diameter, spear head firmness, opening of spear bracts, stem length, flowering time, and spear emergence). Lower Brier scores correspond to greater predictive ability of the model.

To assess the usefulness of the asparagus panel for implementing predictive breeding tools, genotyping data obtained by analysing ddRAD sequencing data were combined with the phenotypic data of eight ordinal categorical traits examined in this study (spear emergence, spear diameter, spear head firmness, opening of bracts, phylloclade density, number of stems, stem length, and flowering time) to fit TGBLUP genomic prediction models using a set of linear predictors, including sex and the effects of molecular markers (Montesinos-López et al., 2015). LOO cross-validation was carried out to estimate the predictive ability of the eight TGBLUP models using BS; this analysis showed that the predictive ability of TGBLUP models ranged from 0.33 for opening of bracts to 0.44 for stem length (**Figure 8**). **Supplementary Figure 3** shows the distribution of probabilities estimated for each categorical phenotype. These probabilities, accounting for the effect of sex, indicated that the average values for class 5 were approximately 0.4 for opening of bracts (0.49), flowering time (0.43), and spear head firmness (0.42), and approximately 0.3 for phylloclade density (0.26) and spear emergence (0.31); for class 2, the average values were approximately 0.3 for number of stems (0.26) and spear diameter (0.3); and finally, for class 6, the average value for stem length was 0.26 (**Supplementary Figure 3**).

Overall, model cross-validation indicated that despite the low number of individuals used to train the models, TGBLUP was capable of predicting these quality traits with high accuracy, highlighting the power of this asparagus collection to implement predictive breeding tools and sustain asparagus breeding.

## Discussion

In the present study, we conducted ddRAD sequencing to monitor molecular diversity in a collection of asparagus genotypes including clones of *A. officinalis*, related wild species, and interspecific lines. Considering the large number of genotypes and the technology used to characterize this genetic material, the present study delivered the most comprehensive survey to date of the available genetic diversity in asparagus using the new generation of molecular markers. A joint analysis of different diploid and tetraploid species was employed to analyze ddRAD sequencing data using a *de novo* strategy to identify genetic variants in order to obtain an unbiased estimate of genetic diversity. Overall, this strategy allowed us to carry out clustering analyses that corroborated previous diversity studies of asparagus based on AFLP fingerprinting or limited sets of SNP markers (Riccardi et al., 2011; Mercati et al., 2013), proving the quality of the SNPs identified using *de novo* analysis.

‘Violetto d’Albenga’ and ‘Morado de Huétor’ are two known populations of tetraploid landraces of asparagus cultivated in Italy and Spain. Molecular analyses carried out to analyze internal transcribed spacers of ribosomal DNA showed that the ‘Morado de Huétor’ population probably originates from an interspecific cross between *A. officinalis* and *A. maritimus* (Moreno et al., 2008), while analysis of phylogeny-based AFLP markers and PCA showed that plants from these two tetraploid populations of landraces cluster together (Riccardi et al., 2011). The panel of asparagus genotypes examined in the present study did not include ‘Morado de Huétor’ plants; nevertheless, PCA corroborated the hypothesis that the ‘Violetto d’Albenga’ population might have originated from an interspecific cross between *A. officinalis* and another asparagus species (**Figure 2**).

Although we used a *de novo* strategy to detect genetic variants, the availability of the asparagus reference sequence allowed us to anchor SNPs detected in diploid genotypes in the reference sequence, which in turn allowed us to provide the first estimate of LD in this species and information regarding genes closely linked to statistically significant SNPs identified in GWAS. LD represents non-random associations between alleles at different loci and reflects several population genetic forces: for instance, it is well known that recombination rate, demographic factors, and the action of selection influence the extent of LD. The analysis of LD presented in this article was carried out in a subset of an asparagus panel containing diploid accessions of *A. officinalis*, most of which were selected for during the course of over 40 years of genetic improvement. Although selection is a known factor that increases LD, the results presented in this article highlight the fact that, in this species, LD decays very rapidly, corroborating the key role of recombination in destroying LD.

### Broadening the genetic bases of cultivated asparagus

Several studies carried out using the first generation of molecular markers (e.g., AFLP) or limited sets of SNPs have indicated the narrow genetic bases of the breeding populations of doubled haploids currently used for the development of hybrid asparagus (Riccardi et al., 2011; Mercati et al., 2015). In the present study, genome-wide SNPs were used to carry out diversity analyses, the results of which corroborated previous findings (**Figures 1**, **2**). The doubled haploids examined in the present study are derived from plants widely used in Italian and international breeding programs over the last 40 years. It is plausible that the genetic erosion observed in this breeding pool of *A. officinalis* is the direct consequence of years of empirical breeding and selection, as has been substantiated in other crops (Van De Wouw et al., 2010). The diversity analysis presented in this article indicates narrow genetic diversity in both cultivated *A. officinalis* and wild accessions of the same species; that is, in our analysis, both groups clustered together (**Figure 2**). In light of this, we conclude that genetic variability within *A. officinalis* L. is very low and other sources of untapped genetic diversity (e.g., interspecific lines) are necessary to sustain breeding for this crop.

The overall analysis of the nine agronomic traits phenotyped in the present study showed that the genotypes included in our asparagus collection predominantly exhibited intermediate values (**Figure 4**). As a large fraction of individuals characterized at the phenotypic levels are doubled haploids of *A. officinalis*, the predominance of few phenotypic classes in the frequency distributions (**Figure 4**) corroborates the narrow genetic bases of these genotypes, although these are preliminary results, and it is necessary to repeat the phenotyping over the course of several years in order to quantify environmental effects on the considered traits.

Although a large fraction of garden asparagus genotypes produce green spears with pink or purple bracts (**Figure 4**; **Supplementary Tables 2**, **3**), the material analyzed in the present study included 25 accessions with entirely green spears, which might be exploited in new breeding programs and might enable study of the regulation of the anthocyanic biosynthetic pathway in asparagus. In contrast, the other phenotypic and agronomic traits examined tended to elicit intermediate scores, with the exceptions of spear diameter and number of stems, for which our material was characterized by rather low values. This is because most of our population was characterized by relatively low levels of heterozygosity (DH and LI), leading them to produce fewer, thinner stems than, for example, commercial hybrids. Interestingly, phenotypic analysis of flowering time variation showed that 40% of the plants fell into intermediate classes.

The correlation between the phylloclade density and the number of stems might highlight that more vigorous plants with a greater number of phylloclades are also able to produce a greater number of stems. Similarly, plants that are not too vigorous but have very long stems could be characterized by lower cladophyll densities, explaining the negative correlation detected in our analysis (**Figure 5**).

In asparagus, proxy traits for plant vigor are number of stalks, heights of the tallest stalks and the first ramification, fresh weight of the fern, number of nodes of the first ramification, and basal diameter of the stalks (Anido et al., 1997). However, a kind of “optical effect” cannot be ruled out: plants with a larger number of stems have a fuller, bushier appearance, so a higher density of phylloclades could also be attributed to them. This visual effect could also lead to inaccurate attribution of a lower density of cladophylls in the case of longer stems, explaining the negative correlation between stem length and phylloclade density.

The analyses carried out in the present study confirm the positive correlation between earliness in spear production and total number of stalks (García et al., 2022). However, unlike these findings, the correlation between the number of spears and spear emergence does not hold in our collection. Correlations between flowering time and number of stems have been highlighted in other studies, although other authors have noted a negative correlation between diameter of the first spear and spear caliber, and have also reported that early yield correlates positively with bush vigor (number and diameter of stems) and early spear diameter (Ellison et al., 1960). Similarly, our results showed a negative correlation between stem length and bract opening: it is known that taller plants (represented by higher values in the present study) exhibit greater branching height, that this trait has a strong positive correlation with spear head tightness (García et al., 2022), and that height of the first ramification, along with stalk diameter, positively correlates with yield (Anido et al., 1997). This is expected, as shoots with poorly closed tips and a high degree of bract opening give rise to shorter plants whose branching starts very close to the ground. Interestingly, no correlation was observed between the opening of bracts and spear head firmness (**Figure 5**). We would have expected a strong negative correlation between these traits, as stems and spears characterized by bract opening have a lower tip closure, and *vice versa*. The low statistical significance of the correlation detected in the present study may be due to the climatic conditions of 2021, which may have strongly influenced one or both traits and consequently their correlation.

### Enabling genome-wide association studies and genome-enabled prediction in asparagus

To date, the GWAS approach has been largely applied in economically important crops, but in asparagus, the identification of the genetic basis of important traits is lagging behind. The creation of the asparagus reference sequence (Harkess et al., 2017) has the potential to foster this type of study. In the present study, we first explored the possibility of carrying out GWAS by applying this methodology to identify markers associated with sex determination. Our GWAS for sex allowed us to identify markers that are very close to genes controlling sex determination in asparagus, corroborating the hypothesis that in asparagus, a limited panel of genotypes might be needed to identify and map markers linked to Mendelian traits if a high density of molecular markers is available. Following these results, we subsequently applied the GWAS method for other agronomic traits identifying markers statistically associated with the anthocyanic coloration of spears, number of stems, and flowering time, and searched for candidate genes in a 60-kb window containing significant markers.

Regarding SNPs associated with asparagus spear coloration, the gene *AsparagusV1_03.907* encodes for a WD repeat-containing protein 82 homologous to *Transparent Testa Glabra1* (*TTG1*). In a study conducted to isolate genes responsible for the initiation of cotton fibers, four putative homologues of the *TTG1* gene regulating *Arabidopsis* trichome development were isolated. Two of these genes also restore anthocyanin biosynthesis in ttg1 mutants of white-flowered *Matthiola incana*. Thus, it is also the case in cotton that homologous *AtTTG1* genes are involved not only in the regulation of trichome development but also in the control of flower coloration (Humphries et al., 2005).

In our analysis, SNP SASPARAGUSV1_04_141920731 was found to be associated with number of stems (**Figure 7**) and was mapped close to *AsparagusV1_04.3450*, which is an expansin-B3-like gene. Expansins are a superfamily that, in plants, are involved in cell wall loosening, cell expansion, and other developmental events during which cell wall modification occurs. Moreover, there is evidence that expansins cause cell wall loosening through a non-enzymatic mechanism, through slippage of the cellulose microfibrils that cover the plant cell wall (Sampedro and Cosgrove, 2005). Expansins are also known to be involved in cell wall modification during nematode infections and are related to plant-type cell wall construction; in a study on laticifers, cells that synthesize and store natural rubber in *Hevea brasiliensis*, expansin-B3-like genes were found to be highly upregulated (Tan et al., 2017). Recently, 241 expansin genes were identified in the wheat genome: wheat expansin genes were found to be differentially expressed in different tissues and organs, suggesting a clear differentiation of functions, and some wheat expansin genes were also found to be significantly upregulated in response to drought and salt stress (Han et al., 2019). Overall, these data corroborate the additional role of the *expansin-B3-like* gene in the mechanisms leading to stem formation in the asparagus plant and its involvement in determining the number of stems.

Among genes close to markers associated with flowering time (SASPARAGUSV1_10_72001201; **Figure 7**), two genes with a subtilisin-like protease function were identified (*AsparagusV1_10.1872* and *AsparagusV1_10.1873*). Subtilisin-like proteases (or subtilases) are a very diverse family of serine peptidases found in many organisms; in plants specifically, they have been shown to play an active role in plant–environment interactions and plant–pathogen communication. Subtilases may also have other biological functions related to protein turnover and to many aspects of the plant life cycle, such as the development of seeds and fruits, cell wall modification, processing of peptide growth factors, and epidermal development (Figueiredo et al., 2018); this evidence leads us to investigate more closely the possible involvement of these two genes (AsparagusV1_10.1872 and AsparagusV1_10.1873) in the processes that regulate flowering time in asparagus. Also present near this SNP is a gene with a myb-related protein 305-like function (*AsparagusV1_10.1876*). These myb-related genes are usually involved in response to abiotic stresses, as in *Vigna unguiculata*, where a myb-related protein 305-like gene has been found to be upregulated after exposure to salt stress (Zhang et al., 2022); however, they can also be associated with fruit production, as in *Vigna mungo*, where a myb-related protein 305-like gene has been found to be associated with a quantitative trait locus (QTL) for yield (Singh et al., 2022), or with the ability to produce giant (mammoth) fruits, as in *Cucurbita maxima* (Reddy et al., 2022). Therefore, it could be reasonable to assume that they may also be involved in determining flowering time and not solely in fruit characteristics.

Overall, considering the relatively limited number of genotypes included in our GWAS and genome-enabled prediction models, we can conclude that the asparagus panel assembled in this study represents a new genomic tool for fostering the dissection of agronomically important traits and the implementation of predictive breeding tools in this crop.

## Data availability statement

The raw sequencing data generated and examined in this article have been deposited in the European Nucleotide Archive under the study number PRJEB60908. Phenotypic data.

## Author contributions

TS: conceptualization, formal analysis, writing—original draft, and supervision. AF: molecular data curation, writing—original draft, and supervision. AL: conceptualization, formal analysis, writing—original draft, and supervision. DP: molecular data curation. LF and FS: phenotypic data curation and plant care. MT: phenotypic data curation and DNA extraction. GLR: supervision. All authors contributed to the article and approved the submitted version.
