# Supplementary material for: Genome-wide analysis of genetic diversity in a germplasm collection including wild relatives and interspecific clones of garden asparagus
Source: Front Plant Sci. 2023 Jul 4;14:1187663. doi: 10.3389/fpls.2023.1187663 (PMC10354869; doi:10.3389/fpls.2023.1187663)
Supplement: Supplementary file 8 [file DataSheet_1.pdf]

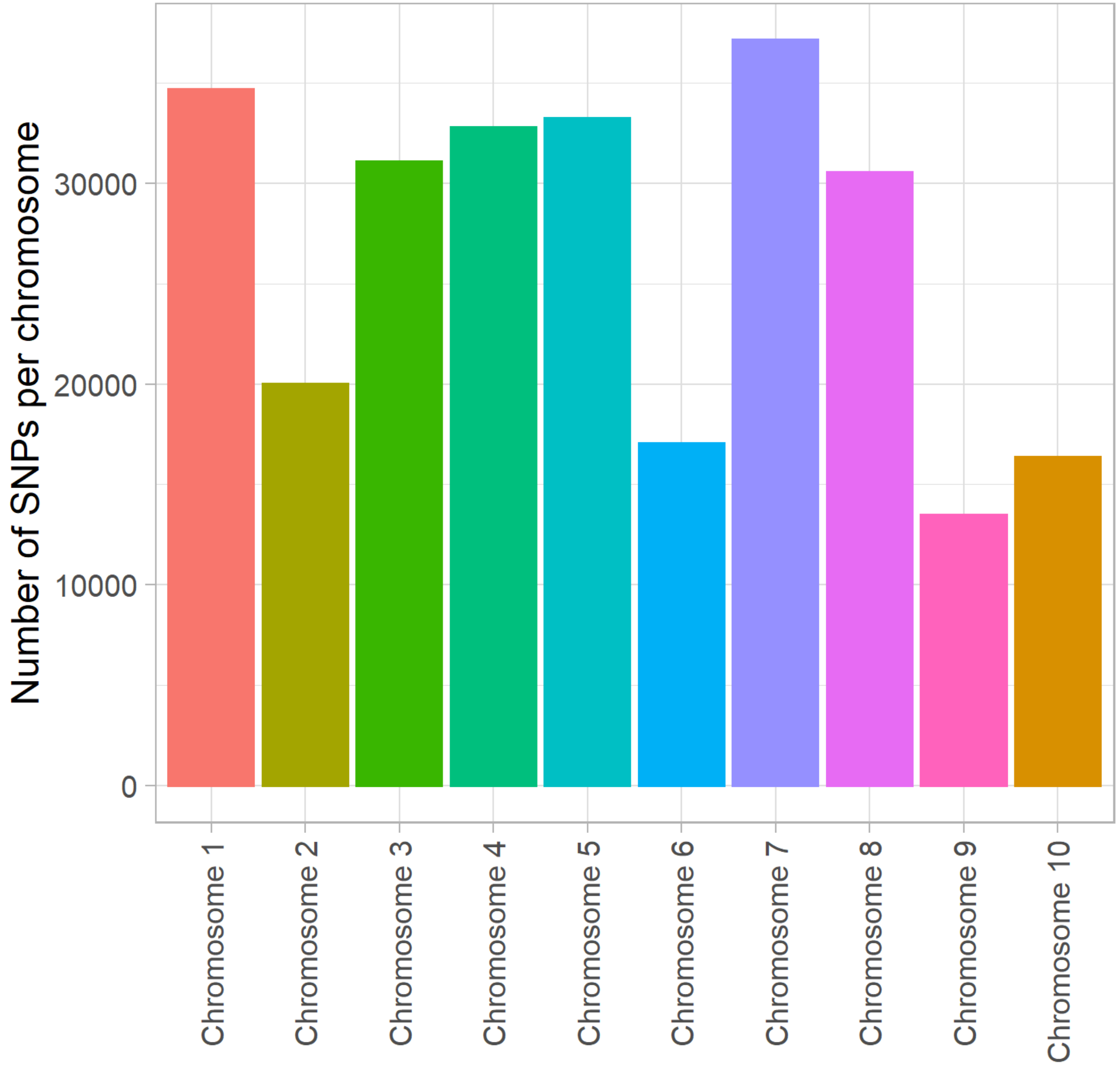

Supplementary Figure 1: Distribution of SNP number across the ten asparagus chromosomes.

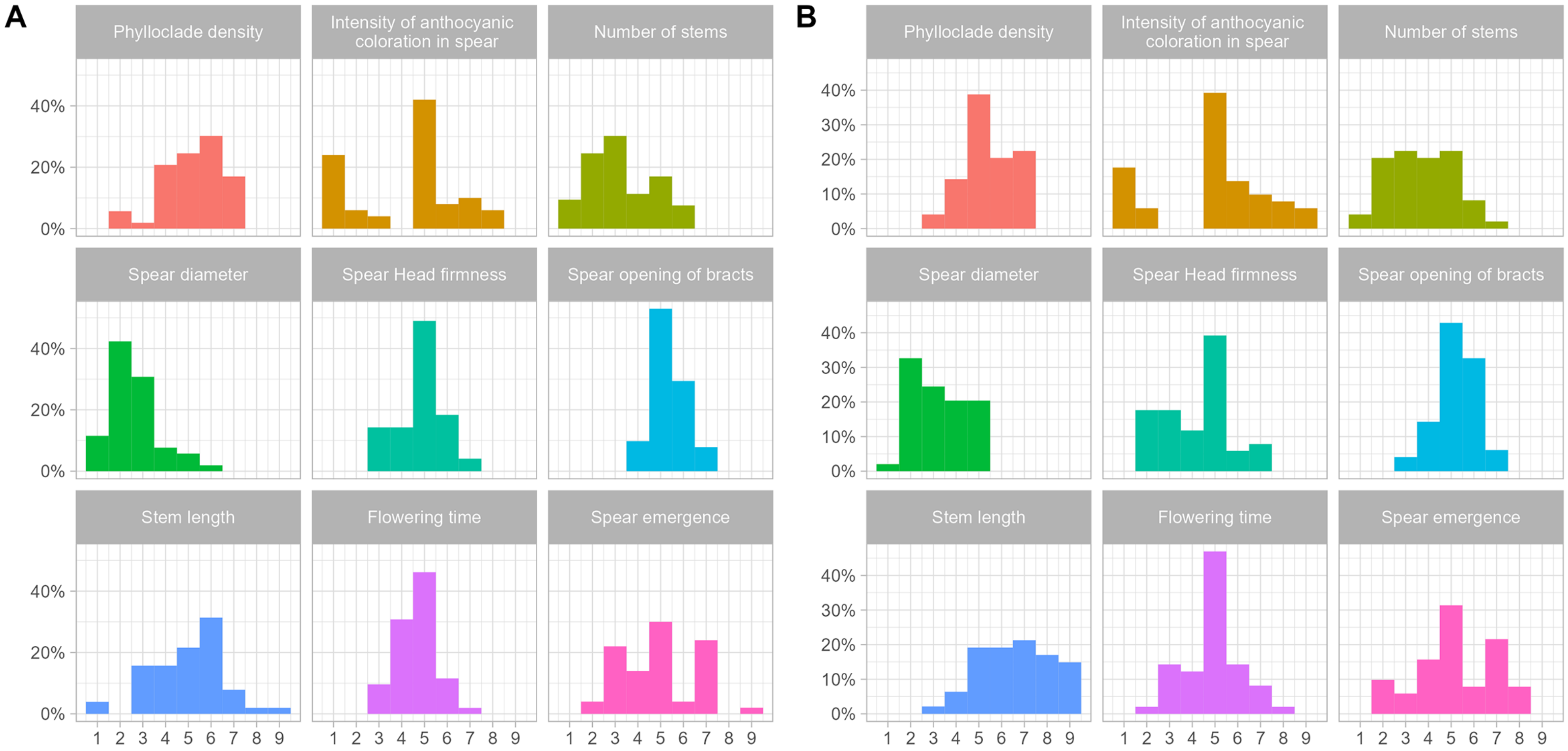

Supplementary Figure 2: Frequency distribution of scores measured in quality and seed production traits. **A)** Histograms show the frequency distribution of nine agronomic traits measured on an ordinal categorical scale varying from 1 to 9 in male plants. **B)** Histograms show the frequency distribution of nine agronomic traits measured on an ordinal categorical scale varying from 1 to 9 in female plants

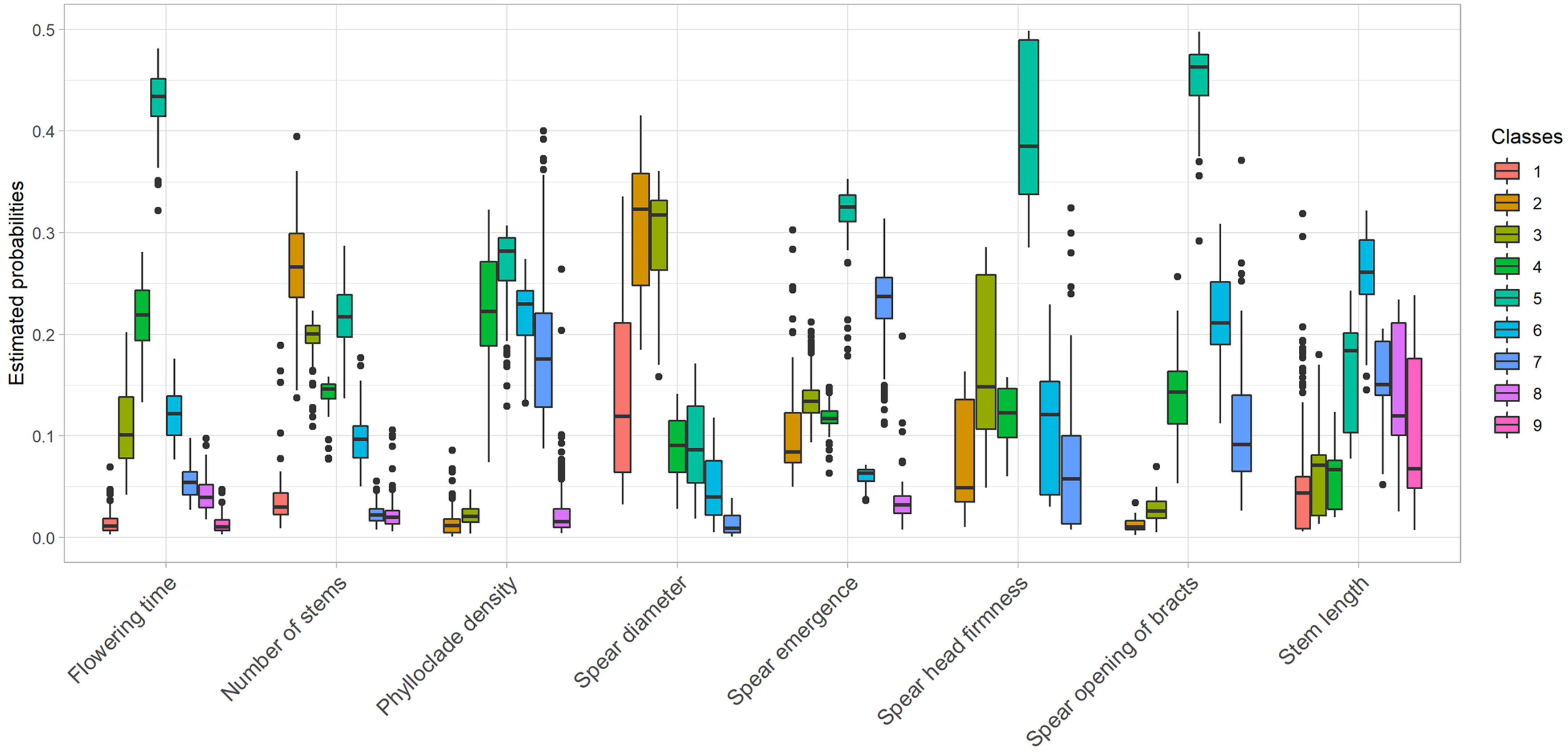

Supplementary Figure 3: Estimated probabilities of genome-enable prediction models for asparagus quality traits. Each boxplot shows the estimated probability in each *Classes* of TGBLUP models carried out for flowering time, number of stems, phylloclades density, spear diameters, spear emergence, spear head firmness, spear opening of bracts and stem length.
